# Integrating holotomography and deep learning for rapid detection of *NPM1* mutations in AML

**DOI:** 10.1038/s41598-024-75168-9

**Published:** 2024-10-10

**Authors:** Hyunji Kim, Geon Kim, HeyJung Park, Mahn Jae Lee, YongKeun Park, Seongsoo Jang

**Affiliations:** 1 Department of Laboratory Medicine, Seoul National University Bundang Hospital, Seongnam, Republic of Korea; 2 Department of Physics, Korea Advanced Institute of Science and Technology, Daejeon, Republic of Korea; 3 KAIST Institute for Health Science and Technology, Korea Advanced Institute of Science and Technology, Daejeon, 34141 Republic of Korea; 4 Department of Laboratory Medicine, Asan Institute for Life Science, Asan Medical Center, Seoul, South Korea; 5 Graduate School of Medical Science and Engineering, Korea Advanced Institute of Science and Technology, Daejeon, Republic of Korea; 6 Department of Laboratory Medicine, Asan Medical Center, University of Ulsan College of Medicine, 88 Olympic-ro 43-gil, Songpa-gu, Seoul, 05505 Republic of Korea; 7 Tomocube Inc., Daejeon, Republic of Korea

**Keywords:** Holotomography, Refractive index, *NPM1*, Deep Learning, Acute myeloid leukemia, Computational biophysics, Acute myeloid leukaemia

## Abstract

Rapid and accurate diagnosis of acute myeloid leukemia (AML) remains a significant challenge, particularly in the context of myelodysplastic syndrome (MDS) or MDS/myeloproliferative neoplasm with *NPM1* mutations. This study introduces an innovative approach using holotomography (HT), a 3D label-free quantitative phase imaging technique, to detect *NPM1* mutations. We analyzed a dataset of 2073 HT myeloblast images from 48 individuals, including both *NPM1* wild-type and mutated samples, to distinguish subcellular morphological changes associated with *NPM1* mutations. Employing a convolutional neural network, we analyzed 3D cell morphology, focusing on refractive index distributions. The machine learning model showed high accuracy, with an area under the receiver operating characteristic curve of 0.9375 and a validation accuracy of 76.0%. Our findings reveal distinct morphological differences between the *NPM1* wild-type and mutation at the subcellular level. This study demonstrates the potential of HT combined with deep learning for early, efficient, and cost-effective diagnosis of AML, offering a promising alternative to traditional stepwise genetic testing methods and providing additional assistance in morphological myeloblast discrimination. This approach may revolutionize the diagnostic process in leukemia, facilitating early detection and potentially reducing the reliance on extensive genetic testing.

## Introduction

The classification of hematologic neoplasms has continually evolved^1–4^ since the first global consensus in 1997^5^, transitioning from a morphology-centric approach to one incorporating genomic data. This evolution reflects a paradigm shift in our understanding of these diseases. Notably, the 2022 revision of acute myeloid leukemia (AML) diagnosis de-emphasizes the traditional 20% leukemic blast cutoff in the presence of driver mutations^3,4^, highlighting the need for comprehensive, yet costly, diagnostic methods for effective treatment planning and risk assessment.

Nucleophosmin (NPM1), a member of the nucleophosmin/nucleoplasmin family, was discovered around 40 years ago^6,7^. Functioning as a nucleo-cytoplasmic shuttling protein, NPM1’s activity is closely linked to its structure, particularly mutations in its C-terminal region, which are crucial in leukemogenesis^8,9^. About 80% of these mutations are type A, characterized by a TCTG duplication in the C-terminal. *NPM1* mutations, found in 30–35% of AML cases^10,11^, were initially identified as a provisional entity in 2008^1^, but were included in the 2017 WHO diagnostic criteria^2^. Their detection methods have evolved from immunohistochemistry to real-time PCR and next-generation sequencing^12^, offering a broader understanding of coexisting genetic alterations.

The mutational status of *NPM1* is vital in guiding treatment decisions, including bone marrow transplantation and specific chemotherapy regimens^4,10,13–15^. The 2024 WHO classification highlight the rapid progression of myelodysplastic syndrome (MDS) or MDS/myeloproliferative neoplasm (MPN) with *NPM1* mutations to AML^16,17^, underscoring the importance of early detection, especially in cases with less than 20% leukemia cells or evidence of multilineage dysplasia. However, identifying *NPM1*-mutated leukemic blasts remains challenging due to the lack of distinct morphological features.

Holotomography (HT), a 3D quantitative phase imaging technique, offers high-resolution, label-free imaging by leveraging refractive index (RI) distributions^18–20^. Functioning as an optical analog to X-ray computed tomography, HT constructs 3D tomograms from multiple 2D holographic images^21–23^. Its advantages include label-free detection, fast assessment, user-friendliness, and the feasibility of objective measurement. HT’s efficacy in differentiating cell types and its integration into deep learning models for advanced screening holds significant promise.

Prior studies have shown the effectiveness of HT in analyzing diverse pathophysiological samples. This includes distinguishing mature cells from blasts^24^, analyzing CD8 + T cells as sepsis biomarkers^23^, and studying biomolecular condensates in bone metastasis^25^. Combined with machine learning approaches^26^, HT has also been effective in classifying lymphocyte subtypes^27^, a task challenging for manual methods. The incorporation of HT data into deep learning models has shown potential in enhancing screening accuracy beyond traditional visual inspection, especially as recent deep learning approaches based solely on image data have demonstrated high sensitivity in classifying bone marrow cells^28^. This study aimed to develop a deep learning model using HT data to predict *NPM1* mutations in leukemic blasts, potentially revolutionizing the diagnostic process for AML.

## Results

### Morphological features of leukemic blasts revealed by holotomography

**Fig. 1:**
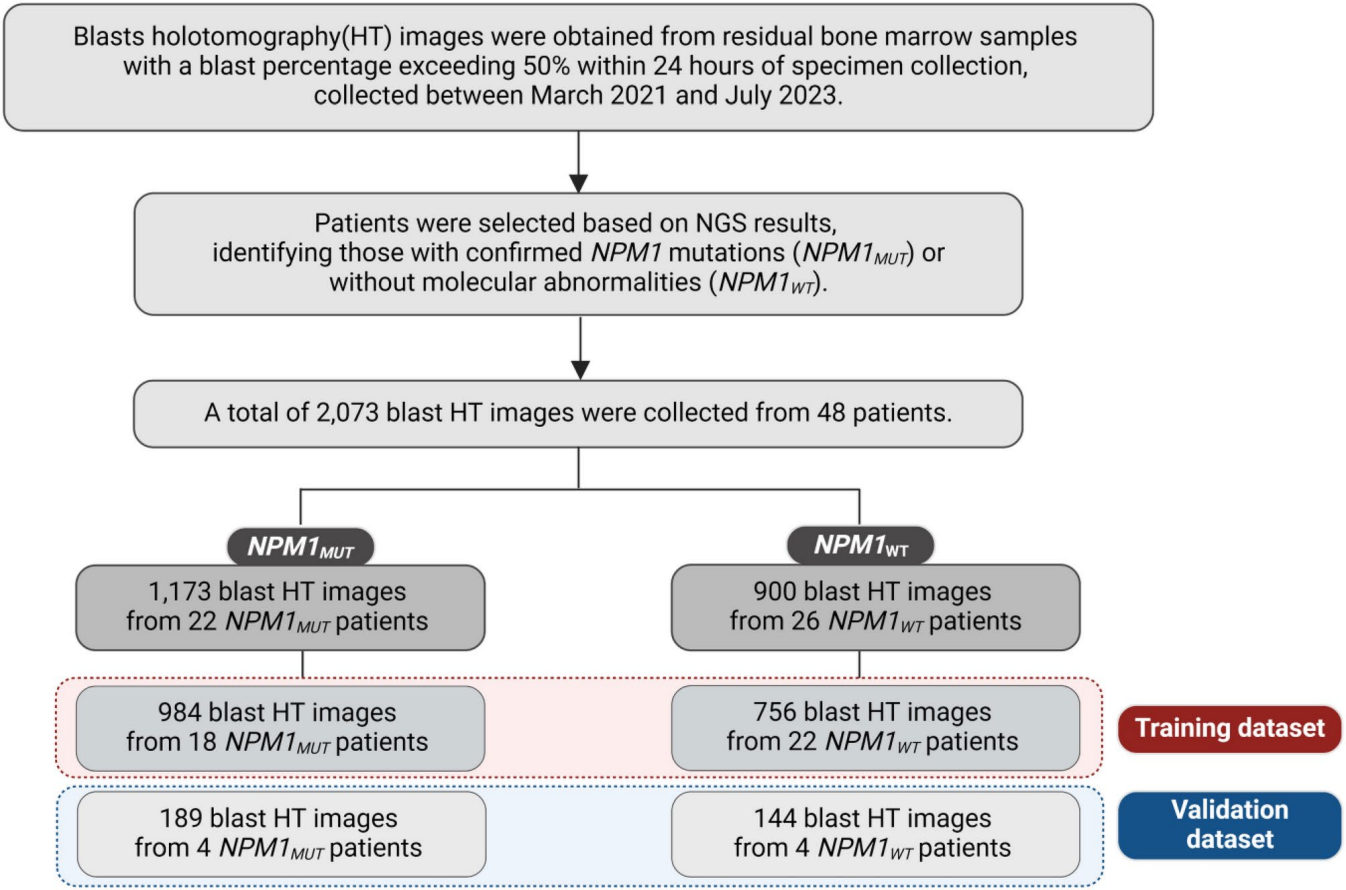
Selection process for the dataset used in the combination of blast holotomography and deep learning to identify alterations related to the NPM1 mutation in leukemic blasts.

**Fig. 2:**
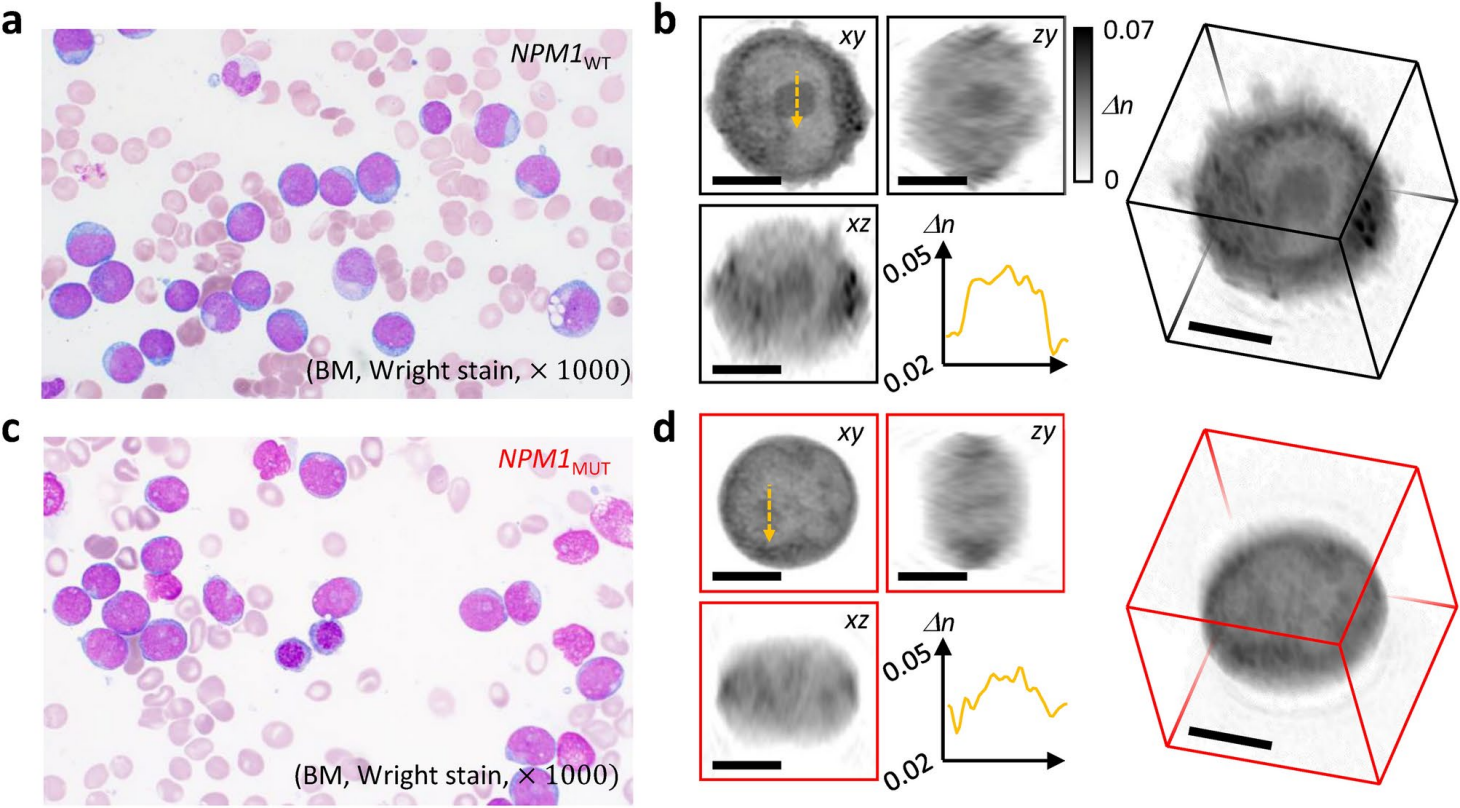
Representative images of myeloblasts from bone marrow (BM) of acute myeloblastic leukemia (AML) patients. (**a**) Wright-stained BM smear image of an *NPM1* wild-type AML patient. (**b**) Myeloblast holographic tomography (HT) image from the same patient, showing central sections in the xy, zy, and xz planes, a line profile of the refractive index through a nucleolus (right), and a three-dimensional rendering of the full volumetric image. (**c**) Wright-stained BM smear image of an *NPM1* mutant AML patient. (**d**) Myeloblast HT image from the same patient, with similar features as in (B).

In our investigation, we analyzed 2,073 HT images of leukemic blasts from 48 specimens. This dataset included 1,173 images from 22 *NPM1*_*MUT*_ specimens and 900 images from 26 *NPM1*_*WT*_ specimens (Fig. 1). The demographic characteristics of the patient groups were similar, as detailed in Table 1. Traditional Wright stain slides did not reveal significant morphological differences between the groups (Fig. 2a,c). However, HT facilitated an in-depth 3D analysis of the leukemic blasts, revealing intricate intracellular RI contrasts (Fig. 2b,d).

**Table 1:** Demographic characteristics and morphological distribution of blast cells in patients by group.

	*NPM1*_*MUT*_ (N = 23)	*NPM1*_*WT*_ (N = 29)	*P*-value
Sex	11:12	14:15	0.975
Age	62.7 ± 13.6	55.6 ± 19.7	0.376
HT Images Count	1173	900	
No. of Images according to morphological classification			
M0	0	129	
M1	886	462	
M2	114	273	
M4	23	9	
M5	150	27

The nuclei of these cells displayed a pronounced nuclear to cytoplasmic ratio (N/C ratio) and were faintly contoured by changes in RI. Within the nuclei, the nucleoli were distinctly visible with defined outlines and typically exhibited a higher RI. The nucleoplasm region showed spatial variations in RI values, possibly reflecting chromatin distribution heterogeneity.

Notably, differences in RI intensity were associated with *NPM1* mutation status. Cells from the *NPM1*_*WT*_ group showed increased RI in the nucleolar regions, while those from the *NPM1*_*MUT*_ group demonstrated comparatively lower RI in these areas. A striking observation was the more defined delineation between the nucleoli and the surrounding nucleoplasm in the *NPM1*_*MUT*_ group. This suggests a potential disruption of the nucleolus structure due to NPM1 protein dislocation, a well-documented consequence of *NPM1* mutations^29^.

In the cytoplasm, cells with *NPM1* mutations exhibited higher RI overall. However, droplets of high RI density were also observed in some *NPM1*_*WT*_ specimens. Additionally, certain HT images of leukemic blasts presented particulate structures with high RI, indicative of a lipid-based composition^30^.

### Numerical parameters of leukemic blasts

**Fig. 3:**
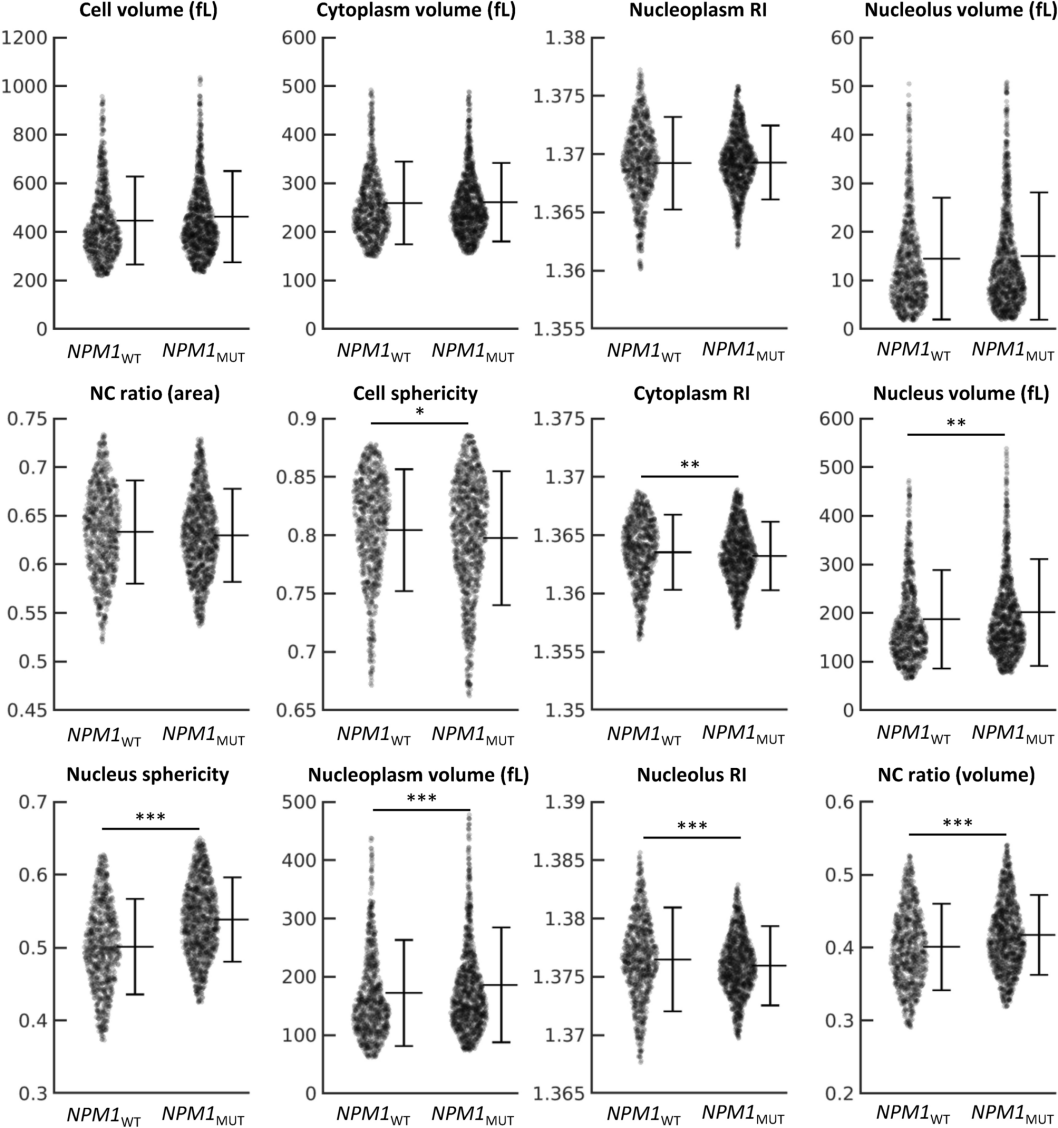
Statistical comparison of myeloblast properties obtained from holotomography (HT) of individual myeloblasts. Each data point represents an individual myeloblast HT image. The average ± standard deviation is displayed on the right of each swarm chart. Data points beyond the 95% confidence interval are not shown to visualize the typical value range better. *, **, and *** denote *P* < 0.01, *P* < 0.001, and *P* < 0.0001 in the Mann–Whitney U test. The absence of any asterisk indicates no significance.

Our study identified 39 distinct numerical parameters associated with leukemic blasts, previously undocumented and detailed in Supplementary Table 1. Comparative analysis of these parameters revealed significant differences in cell and nucleus sphericity, nucleolus volume, N/C ratio, and RI values of the cytoplasm and nucleolus between the *NPM1*_*WT*_ and *NPM1*_*MUT*_ groups (Fig. 3).

Regarding cell volume, *NPM1*_*MUT*_ blasts demonstrated a slightly larger average volume (462.18 ± 187.43 fL) compared to *NPM1*_*WT*_ blasts (446.24 ± 181.18 fL), although this difference was not statistically significant. Similar marginal distinctions were observed in nucleolus volume (*NPM1*_*WT*_: 14.48 ± 12.54 fL, *NPM1*_*MUT*_: 14.99 ± 13.09 fL), cytoplasm volume (*NPM1*_*WT*_: 259.28 ± 84.73 fL, *NPM1*_*MUT*_: 260.90 ± 80.70 fL), and nucleoplasm RI (*NPM1*_*WT*_: 1.3692 ± 0.0039, *NPM1*_*MUT*_: 1.3692 ± 0.0031).

Notably, *NPM1*_*MUT*_ blasts exhibited significantly reduced cell sphericity (*NPM1*_*WT*_: 0.804 ± 0.052, *NPM1*_*MUT*_: 0.797 ± 0.057) and lower cytoplasm RI (*NPM1*_*WT*_: 1.3635 ± 0.0032, *NPM1*_*MUT*_: 1.3632 ± 0.0029). The nucleus volume in *NPM1*_*MUT*_ blasts was also larger (*NPM1*_*WT*_: 186.71 ± 101.13 fL, *NPM1*_*MUT*_: 201.07 ± 109.97 fL). More pronounced differences were evident in nucleus sphericity (*NPM1*_*WT*_: 0.501 ± 0.065, *NPM1*_*MUT*_: 0.538 ± 0.057), nucleoplasm volume (*NPM1*_*WT*_: 172.22 ± 90.94 fL, *NPM1*_*MUT*_: 186.08 ± 98.36 fL), nucleolus RI (*NPM1*_*WT*_: 1.3764 ± 0.0044, *NPM1*_*MUT*_: 1.3759 ± 0.0034), and NC volume ratio (*NPM1*_*WT*_: 0.400 ± 0.059, *NPM1*_*MUT*_: 0.417 ± 0.054). Intriguingly, the N/C ratio difference was only appreciable in the 3D analyses, as opposed to the 2D projected area (*NPM1*_*WT*_: 0.633 ± 0.053, *NPM1*_*MUT*_: 0.629 ± 0.047). These observed morphological changes, particularly the spherical inflation of the nucleus, disruption of nucleolar aggregation, and alteration in cytoplasmic roundness, are hypothesized to correlate with the dislocation of the mutated NPM1 protein.

### Artificial-intelligence-assisted prediction model of NPM1 mutation: classifier development and validation

We leveraged HT combined with deep learning to identify mutation-related alterations in leukemic blasts. Our deep learning classifier was trained on 1,740 HT images of blasts from 40 patients and validated using an independent dataset of 333 HT images from eight patients. The training dataset consisted of 984 images from 18 *NPM1*_*MUT*_ patients and 756 images from 22 *NPM1*_*WT*_ patients. The validation dataset included 189 images from four *NPM1*_*MUT*_ patients and 144 images from four *NPM1*_*WT*_ patients (Fig. 1).

**Fig. 4:**
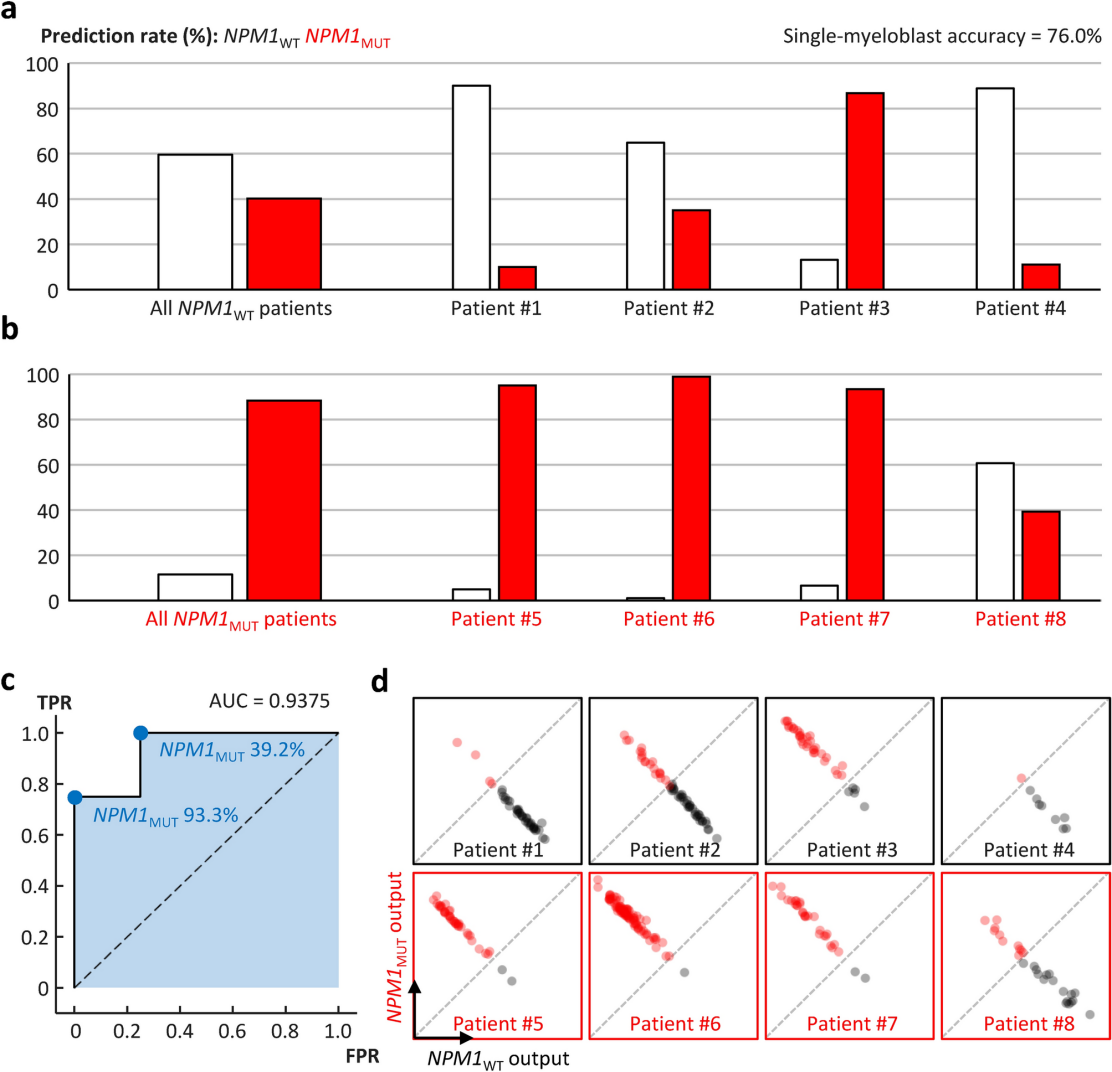
Deep learning classification of myeloblast holotomography (HT) images for *NPM1* mutation status. (**a**) Validation result of the deep learning classifier on myeloblast HT images of four *NPM1* wild-type patients. (**b**) Validation result of the deep learning classifier on myeloblast HT images of four *NPM1* mutant patients. (**c**) Receiver operating characteristic curve representing the performance of patient-wise *NPM1* mutation screening based on different threshold positive prediction ratios. (**d**) Plot of deep learning output values corresponding to the two classes (wild-type and mutant). Each data point represents an individual myeloblast HT image. The dashed diagonal line indicates the boundary where the two output values are equal.

**Fig. 5:**
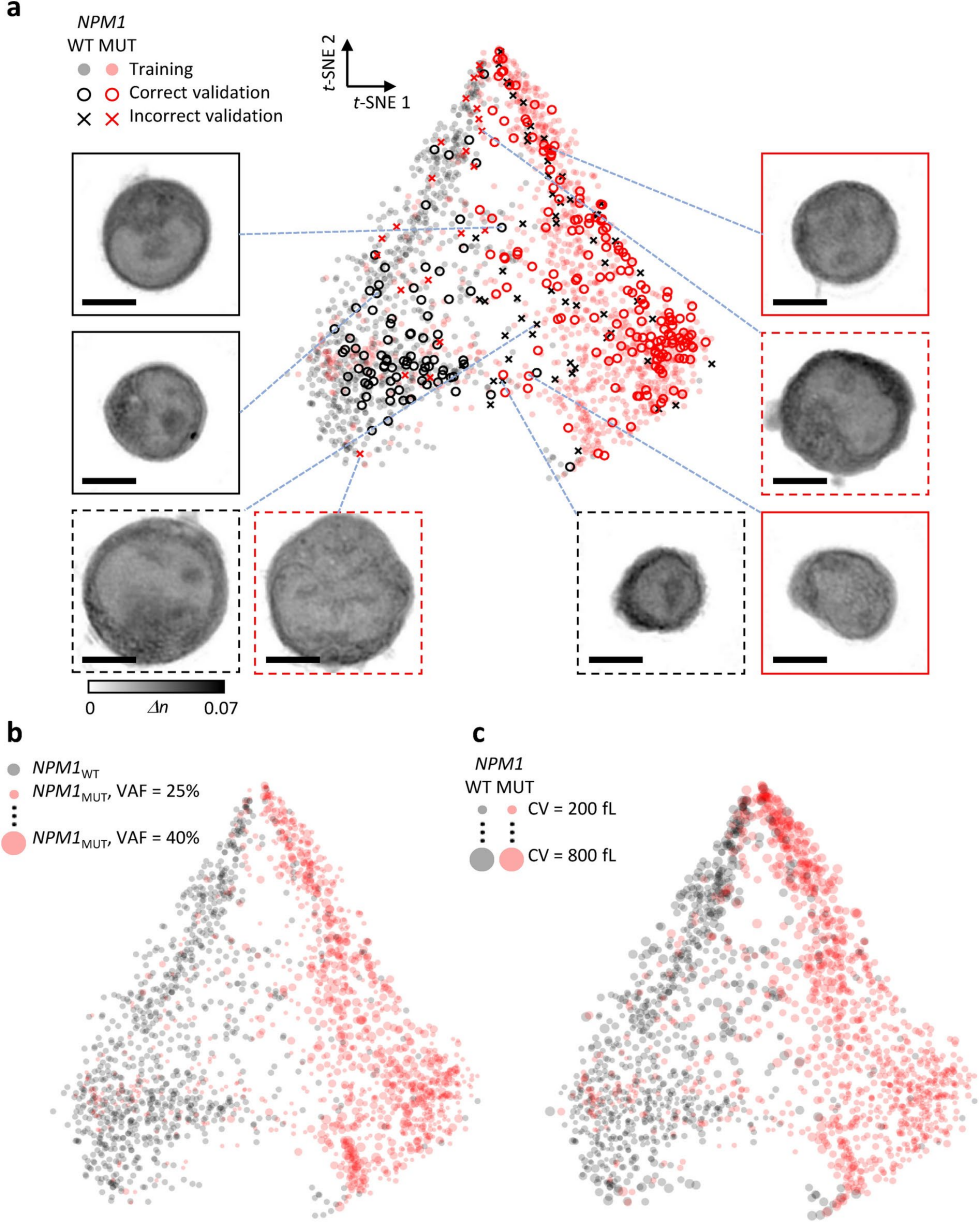
Latency of 264 dimensions visualized in 2D using t-SNE. (**a**) Validation data marked based on prediction accuracy, depicting consistent feature extraction and error distribution. Central sections of error cases shown in various regions of latent feature space. Scale bar = 5 µm. (**b**) *NPM1* mutant data marked with size dependent on variant allele frequency, showing moderate correlation with mutation degree. (**c**) All data marked with size dependent on cell volume, illustrating size-dependent clustering of myeloblast data.

In the validation phase, the classifier accuracy for single blast HT images was 76.0%. Misclassification rates were 20.4% for *NPM1*_*WT*_ and 25.8% for *NPM1*_*MUT*_ blasts (Fig. 4a,b). The t-SNE analysis demonstrated a clear separation of latent features learned by the classifier between *NPM1*_*MUT*_ and *NPM1*_*WT*_ (Fig. 5). Notably, the classifier exhibited strong correlation between the training and validation datasets within both the *NPM1*_*MUT*_ and *NPM1*_*WT*_ groups, underscoring its robustness. Importantly, this correlation is significant because the training and validation data were independently acquired from distinct patient cohorts, highlighting the classifier’s ability to generalize across different datasets by focusing on HT features relevant to *NPM1* mutations.

However, some misclassifications occurred, particularly in cells with atypical sizes or poorly defined nuclear boundaries (Fig. 5a). Additional analyses revealed key insights into the classifier’s decision-making process, including spatial feature mapping for each class (Supplementary Fig. S1). While the current network design outperformed lighter variants in our trials (Supplementary Fig. S2), these alternatives may offer benefits such as faster training times and reduced overfitting. Moreover, the high 3D resolution provided by HT was found to enhance single-blast accuracy, as reducing data resolution led to diminished performance (Supplementary Fig. S3).

### Insights from t-SNE mapping and ROC analysis

Further t-SNE mapping correlated misclassifications with variant allele frequency (VAF) (Fig. 5b) and cell volume (Fig. 5c). Misclassified cases and those outside the *NPM1*_*MUT*_ prediction lobe typically showed low VAF, and cells with abnormally large volumes were more common in the apical zones of both the *NPM1*_*WT*_ and *NPM1*_*MUT*_ lobes.

To evaluate the classifier’s effectiveness in determining the *NPM1* genotype in the validation patients, we employed the receiver operating characteristic (ROC) curve by varying the threshold of the *NPM1*_*MUT*_ prediction rate (Fig. 4c). The optimal true positive rate (TPR) with a zero false positive rate (FPR) was 0.75 at a threshold of 93.3%. Conversely, the lowest FPR with a TPR of 1.0 was 0.25 at a threshold of 39.2%. The area under the ROC curve was 0.9375.

An analysis of classifier outputs revealed that variations in prediction across patients resulted from overall shifts in classifier outputs rather than individual outliers (Fig. 4d). The distribution of outputs for both genotypes showed trends toward the predicted genotype, with the extent of the shift correlating with the prediction rate.

## Discussion

**Fig. 6:**
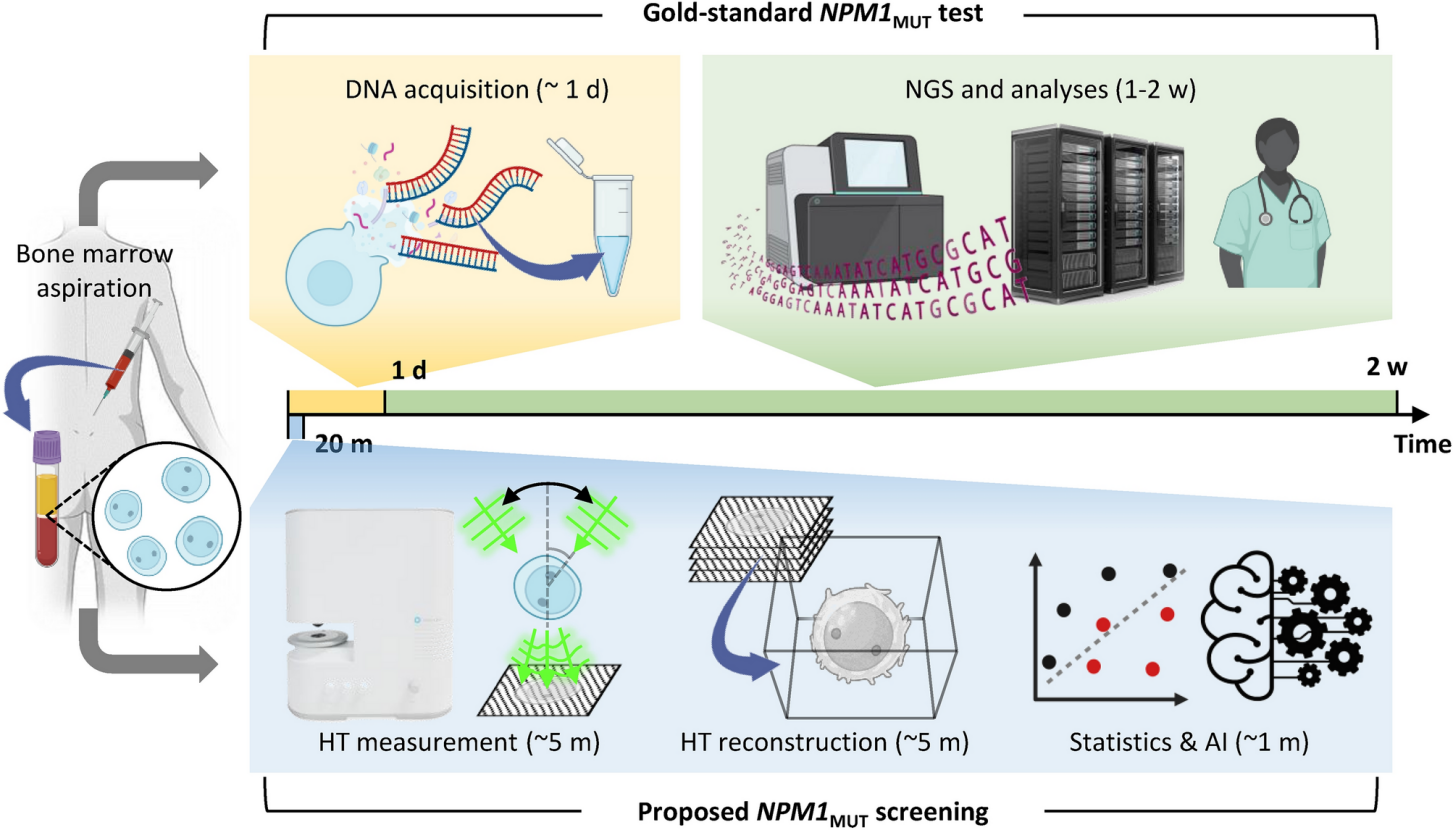
Schematic illustration of the proposed *NPM1* mutation screening method in comparison to the gold standard test. The gold standard test provides the confirmatory genetic profile based on DNA next-generation sequencing, yet the typical turnaround time may stretch to 2 weeks. The proposed method can provide an early screening based on the three-dimensional morphology of myeloblasts using holotomography (HT) imaging aided by statistical and artificial intelligence analyses.

The morphological overlap among leukemic blasts often hinders differentiation using conventional optical microscopy. However, HT and AI have shown promise in identifying *NPM1* mutations, with an accuracy of 76.0% in single-cell mutation determination and an AUC of 0.9375 for patient-wise detection. HT’s unique capability in 3D label-free imaging, coupled with deep learning image classification, provides a rapid and cost-effective method for detecting *NPM1* mutations in AML patients. The entire process, from cell imaging to AI-driven final analysis, was completed within a mere 20 min with no requirements for the use of experts or exogenous labeling agents (Fig. 6).

HT’s key advantages include minimal examination costs and the ability to confirm cellular RI images while calculating numerical data. The RI features of the nucleoli and cytoplasm are particularly pronounced in *NPM1*_*MUT*_ blasts, facilitating their identification. This observation is supported by IHC staining, which confirms protein dislocation associated with *NPM1* mutations^10^. HT’s sensitivity in detecting minute protein changes (as subtle as 5.4 fg/fL with a noticeable RI difference of 0.001)^31^ and its linear correlation between the RI value and protein concentration^32,33^ allow for precise quantification of protein and cellular alterations due to genetic mutations.

Our study’s numerical parameter analysis indicates that *NPM1* mutations cause spherical nuclear inflation, nucleolar aggregation disruption, and cytoplasmic morphology deformation^11^. These alterations, previously observed with anti-N-terminal antibody staining, are more pronounced in three-dimensional views. In two-dimensional optical microscopy, these changes mimic monoblast-like morphology, suggesting characteristics of FAB M4 or M5 subtypes in AML with mutated *NPM1*^2^.

The integration of AI with an HT database focused on detecting various genetic alterations in leukemic blasts is crucial for objective and precise cell classification. As molecular genetic alterations play an increasingly important role in predicting patient outcomes, especially as the traditional 20% blast threshold for leukemia diagnosis becomes less definitive, this approach has significant potential^34–36^. In resource-limited settings, where advanced molecular tests like NGS may not be routinely performed, crucial diagnoses may be missed. For instance, if the blast count exceeds 10%, the absence of molecular testing could lead to incorrect or delayed treatment decisions. Our approach addresses this gap by enabling early mutation detection in hematologic malignancies, facilitating timely and accurate classification. This not only expedites the identification of cases requiring further genetic testing but also ensures that patients receive the appropriate treatment for their specific disease state, optimizing the allocation of medical resources.

The limitations of our study include the relatively small validation dataset, which may affect the generalizability of our findings. Future studies with larger patient cohorts are necessary to enhance the method’s robustness and reliability, as indicated in our comparative study with reduced training data (Supplementary Fig. S4). Additionally, the genetic heterogeneity of AML introduces significant challenges related to inter-patient variability. Even within individual patients, somatic mutation changes can vary depending on the specific clone producing each cell^37–39^. This diversity is well-established and is associated with various prognoses based on coexisting gene mutations, particularly among AML patients with mutated *NPM1*^4,40^. While a dominant clone within a one patient may exhibit similar characteristics, as demonstrated in our study, challenges in achieving higher accuracy may arise due to associated morphological changes among patients.

To improve early screening of leukemic blasts that require urgent assessment, it is critical to train AI models on a wider variety of AML blasts encompassing diverse diagnostic classifications. This will enhance the effectiveness of screening tools. Moreover, as data volume increases, future studies will require improvements in both software and hardware. With our current setup—comprising the network design, input pipeline, and hardware—200 training iterations take 36 h and utilize 30.96 GB of video memory, while each set of network parameters requires 925.30 MB of storage. Although feasible on a high-performance personal computer, larger databases in future studies may necessitate more efficient software or advanced computational resources. Software enhancements, such as refining the loss function and incorporating data augmentation, offer promising strategies to better leverage large-scale data. Additionally, future research should focus on more finely stratified genetic subgroups to improve the differentiation of morphological features and mitigate variability within the AML patient population.

In conclusion, our work underscores the potential of combining HT and AI for early *NPM1* mutation detection, offering a complementary tool to traditional diagnostic methods. The integration of this approach with genetic confirmation and prognostic analysis will bolster its utility in AML diagnosis. Despite current limitations, our promising results lay the groundwork for further research aimed at refining and expanding this methodology, contributing to more effective AML diagnostic strategies.

## Methods

### Specimen selection

Between March 2021 and July 2023, our research focused specifically on utilizing residual specimens obtained from patients who underwent bone marrow (BM) examinations. The process of specimen selection for analysis unfolded in three distinct stages.

In the first stage, residual BM specimens were characterized by a leukemic blast percentage that exceeded 50%. Utilizing HT-1H (Tomocube Inc., Daejeon, Republic of Korea), we captured images and numerical indices of leukemic blasts within 24 h of specimen collection.

Moving to the second stage, the procedure involved filtering specimens based on the results of NGS. Specifically, individuals with confirmed *NPM1* mutations identified in the NGS results were designated as the experimental group (Mutated *NPM1*, *NPM1*_*MUT*_). Specimens lacking molecular abnormalities associated with AML defined by genetic abnormalities or AML with recurrent genetic abnormalities were selected as potential specimens for the control group (Wild-type *NPM1*, *NPM1*_*WT*_).

In the third stage, two pathologists conducted a meticulous examination of the Wright-stained slides of the selected specimens for the final selection of the *NPM1*_*WT*_ group. By comparing the morphology of leukemic blasts in the *NPM1*_*MUT*_ group specimens, those with visually indistinguishable features were selected for the *NPM1*_*WT*_ group.

### HT imaging system and numerical parameter extraction

We utilized a laser-based holotomography system (HT-1H, Tomocube Inc., Daejeon, Republic of Korea). To capture individual images of leukemic blasts, residual BM specimens were diluted in a phosphate-buffered saline (PBS) solution, adjusted to the physiological pH of the living organism^41,42^. Then, we place 20 μL of the diluted specimen, on a 25 × 50 mm cover slide (C025501, Matsunami Glass Ind., Japan), covered it with another cover slide, and conducted the measurements.

During cell measurements, the HT-1H microscope not only provides visualization of the cells but also enables the measurement of the RIs of various intracellular structures (For more information, refer to the Supplementary Text.). Through this, it allows for the generation of numerical data pertaining to spatial information and physical characteristics. The image reconstruction was performed with TomoStudio 2.7.3 (Tomocube Inc., Daejeon, Republic of Korea), specialized software for the measurement and processing of the HT-1H data.

The cellular properties were calculated from each HT image using the linear relationship between the biomolecule concentration and the RI contrast, along with image segmentation for subcellular regions. The properties of the entire cell or an intracellular region include volume, surface area, sphericity, average RI, and dry mass.

The inter-group difference in the distribution of properties was assessed using the Mann–Whitney U test due to uneven sample sizes and the necessity for a non-parametric approach to the distributions. For subcellular region segmentation, we utilized ilastik 1.4.0^43^, a machine learning pixel classification software, followed by numerical post-processing based on priors about cellular morphologies. The numerical processing and analyses were performed using MATLAB 2022a (MathWorks, Natick, MA).

### Deep learning classifier and optimization

For deep learning image classification, a convolutional neural network (CNN) designed for multi-resolution feature preservation was implemented. This CNN architecture, inspired by FishNet^44^, comprises three primary components—the tail, body, and head—in the feed-forward order. Additionally, it includes initial convolutional layers, tail-body bridging layers, as well as classifying layers. The tail, body, and head components extract features while downsampling, upsampling, and downsampling, respectively. A 2 × 2 × 2 max-pooling operation downsamples the feature map, whereas a 2 × 2 × 2 nearest-neighbor resizing operation upsamples the feature map. A distinct mechanism of this architecture is the recollection of equal-sampling features from the shallower layers. For instance, the feature maps after each upsampling operation in the body layer are concatenated with the first equal-sampling feature maps of the tail layer.

For a more quantitative description of the CNN, it includes a total of 80,839,106 adjustable parameters, mostly accounted for by the 247 convolutional layers. Residual bottleneck blocks^45^ precede every downsampling or upsampling operation. Each residual block results in a summation of two feature maps: one through a single 1 × 1 × 1 convolutional layer and the other through a series of 1 × 1 × 1, 3 × 3 × 3, and 1 × 1 × 1 convolutional layer that reduce the number of feature map channels. Every convolutional layer is preceded by instanceNorm^46^ and leaky ReLU pre-activation^47^. A more graphical description of the CNN classifier can be found in Supplementary Fig. S5.

The parameters of the CNN were optimized through gradient-based reduction of the cross-entropy loss function. The cross-entropy loss was weighted inversely proportional to the class-specific size of the training dataset to evenly penalize errors despite the class imbalance in the training dataset. The Adam optimizer was employed to adjust the parameters with the following hyperparameters: an initial learning rate of 0.0005, learning rate decay of 0.0001, gradient moment decay of 0.5, and second-order gradient moment decay of 0.999. The learning rate was further modulated through cosine annealing with a period of 32 to facilitate the exploration of a wide range of parameters. The batch size was 16, and each HT image of the training dataset was fed twice—once in its original form and once in a randomly augmented form—during each iteration through the dataset. Augmentation included the addition of Gaussian noise, random translation, horizontal flipping, and rotation. The size of each input HT image was 160 × 160 × 72.

The optimization process ran on a computational workstation, utilizing four graphics processing units of GeForce GTX 1080 Ti (NVIDIA Corporation, Santa Clara, CA) and eight workers from an Intel Xeon E5-2630 processor (Intel Corporation, Santa Clara, CA). In this hardware setting, each iteration through the training dataset consumed approximately 6 min. The training accuracy of the CNN saturated within 200 iterations. The CNN and its optimization process were implemented in a Python 3.7.16 environment that included the deep learning library Torch 1.0.0.

The final parameters of the CNN classifier were chosen based on the performance in the training dataset. To be specific, a portion of the training dataset with the size comparable to the validation dataset was used to assess the generalization accuracy instead of directly reflected during optimization. The quantities of optimization data is 637 and 574 for *NPM1*_*WT*_ and *NPM1*_*MUT*_ respectively. The quantities of generalization data is 144 and 410 for *NPM1*_*WT*_ and *NPM1*_*MUT*_ respectively. Among different sets of the CNN parameters yielded from the 200 iterations, the one with the highest sum of training accuracy and generalization accuracy was selected.

For the investigation of deep learning features, a 264-dimensional feature was extracted from each HT image prior to the final output layer of the CNN. After reducing the dimensions through t-distributed stochastic neighbor embedding (t-SNE), a 2D visualization was performed. The optimization process of t-SNE was conducted under a learning rate of 200 and a perplexity of 200, after standardizing each feature dimension to have 0 average and 1 standard deviation. The embedding and visualization were carried out using MATLAB 2022a (MathWorks, Natick, MA, USA).

## Supplementary Information

Supplementary Information.

## Data Availability

The datasets used and/or analyzed during the current study are available from the corresponding author on reasonable request.
