# Supplementary material for: Integrating holotomography and deep learning for rapid detection of NPM1 mutations in AML
Source: Sci Rep. 2024 Oct 10;14:23780. doi: 10.1038/s41598-024-75168-9 (PMC11467337; doi:10.1038/s41598-024-75168-9)

Supplementary text. Principle of Holotomography

Positioned on the sample illumination path, the digital micromirror device (DMD) served as a controllable binary grating, enabling the alteration of the illumination angle. By adjusting the spatial frequency of the periodic pattern on the DMD, the illumination angle could be scanned, resulting in an angular series of 2D holographic measurements. Each sample's measurement included a total of 49 illumination angles, covering both a normal angle and 48 oblique angles uniformly distributed in the azimuthal direction.

Subsequently, the 3D refractive index (RI) tomogram underwent reconstruction from the sinogram, following the principles of optical diffraction tomography. This reconstruction involved solving the inverse Helmholtz equation, followed by an iterative regularization process to address the missing cone problem.

The light source employed was a continuous-wave laser with a wavelength of 532 nm. Two water-immersion objective lenses with a numerical aperture of 1.2 were used for magnification and de-magnification. The polar angle of the oblique illumination corresponded to a numerical aperture of 0.9. The theoretical resolution of the tomograms was calculated at 110 nm horizontally and 330 nm vertically, considering the spatial frequency range of the imaging system.

The measurement of an entire sinogram required approximately 0.4 s, primarily constrained by the camera frame rate. The reconstructed images were resampled into a voxel resolution of 96 × 96 × 192 nm for visualization and further analyses.

Supplementary Table 1. Intergroup comparison results of numerical parameters calculated from HT images of leukemic blasts.

Numerical parameter	*NPM1*$_{MUT}$	*NPM1*$_{WT}$	*P*-value
Cell Otsu threshold†	13521.55762 ± 15.16038	13521.37207 ± 18.19734	0.228886
Cell volume (fL)	462.18436 ± 187.43539	446.24902 ± 181.18277	0.014599
Cell surface area (mm^2)	363.57138 ± 119.23312	351.00168 ± 112.63622	0.007065
Cell RI mean	1.36595 ± 0.00293	1.36606 ± 0.003	0.020185
Cell dry mass (pg)	71.36263 ± 27.17676	68.87076 ± 25.58730	0.028932
Cell dry mass density (g/dL)	0.15648 ± 0.01584	0.15709 ± 0.01888	0.020191
Cell RI SD	0.00861 ± 0.00089	0.00875 ± 0.00097	<0.05
Cell RI entropy	0.70680 ± 0.16871	0.74295 ± 0.16740	0.000001
Cell sphericity	0.79746 ± 0.05744	0.80431 ± 0.05217	0.00605
Cell projected area (mm^2)	80.05155 ± 26.29934	79.31763 ± 26.59682	0.420318
NC volume ratio‡	0.41750 ± 0.05495	0.40094 ± 0.05924	<0.05
Nucleoplasm volume (fL)	186.08298 ± 98.36874	172.22986 ± 90.94092	0.000041
Nucleoplasm RI mean	1.36927 ± 0.00318	1.36922 ± 0.00398	0.667537
Nucleoplasm RI SD	0.00490 ± 0.00091	0.00510 ± 0.00093	<0.05
Nucleoplasm RI entropy	0.52394 ± 0.28956	0.55039 ± 0.31090	0.036035
Nucleoli volume (fL)	14.99619 ± 13.09568	14.48031 ± 12.54512	0.739559
Nucleoli RI mean	1.37595 ± 0.00340	1.37649 ± 0.00445	0.000006
Nucleoli dry mass (pg)	3.13837 ± 2.74365	3.07851 ± 2.69303	0.992705
Nucleoli RI SD	0.00394 ± 0.00102	0.00406 ± 0.00097	0.000637
Nucleoli RI entropy	0.77920 ± 0.25529	0.68790 ± 0.29472	<0.05
Cytoplasm volume (fL)	260.90414 ± 80.70547	259.28403 ± 84.73240	0.280815
Cytoplasm RI mean	1.36321 ± 0.00294	1.36353 ± 0.00322	0.000124
Cytoplasm dry mass	36.30492 ± 9.37407	36.34980 ± 9.45662	0.70694
Cytoplasm RI SD	0.00943 ± 0.00108	0.00954 ± 0.00106	0.000005
Cytoplasm RI entropy	0.74520 ± 0.11678	0.77355 ± 0.10634	<0.05
LD volume (fL)	0.20103 ± 0.76623	0.25485 ± 0.51597	<0.05
LD RI	1.37116 ± 0.02352	1.38160 ± 0.01613	<0.05
LD dry mass (pg)	0.07674 ± 0.29760	0.10010 ± 0.21339	<0.05
LD RI SD	0.00303 ± 0.00305	0.00453 ± 0.00307	<0.05
LD RI entropy	0.20229 ± 0.32883	0.31079 ± 0.36856	<0.05
Nucleoplasm PA	51.17463 ± 20.33812	51.01690 ± 20.44743	0.822253
Nucleoli PA	11.37256 ± 7.56295	11.22828 ± 7.43448	0.870592
Cytoplasm PA	80.05016 ± 26.29848	79.31589 ± 26.59520	0.420489
LD PA	0.33122 ± 0.95428	0.41007 ± 0.69808	<0.05
NC Ratio (PA)‡	0.62987 ± 0.04780	0.63328 ± 0.05300	0.022931
Nucleus PA	51.17463 ± 20.33812	51.01690 ± 20.44743	0.822253
Nucleus Volume	201.07915 ± 109.97424	186.71022 ± 101.13725	0.000199
Nucleus Surface Area	301.14020 ± 103.62543	309.30511 ± 109.23205	0.085862
Nucleus Sphericity	0.53853 ± 0.05768	0.50138 ± 0.06557	<0.05

†RI value as a standard when distinguishing between background and cell using Otsu's method, ‡Calculate nucleus-cytoplasmic ratio by volume or area

- Abbreviations: RI, refractive index; SD, standard deviation; NC ratio, nucleus to cytoplasmic ratio; PA, projected area.

Supplementary Figure S1. Visualization of classification-related features. (a) Representative Grad-CAM map of an *NPM1*$_{WT}$ blast. (b) Representative Grad-CAM map of an *NPM1*$_{MUT}$ blast. (c) The average Grad-CAM level in different subcellular regions of a blast. The distribution of Grad-CAM level is separately plotted based on the ground truth class and the prediction.

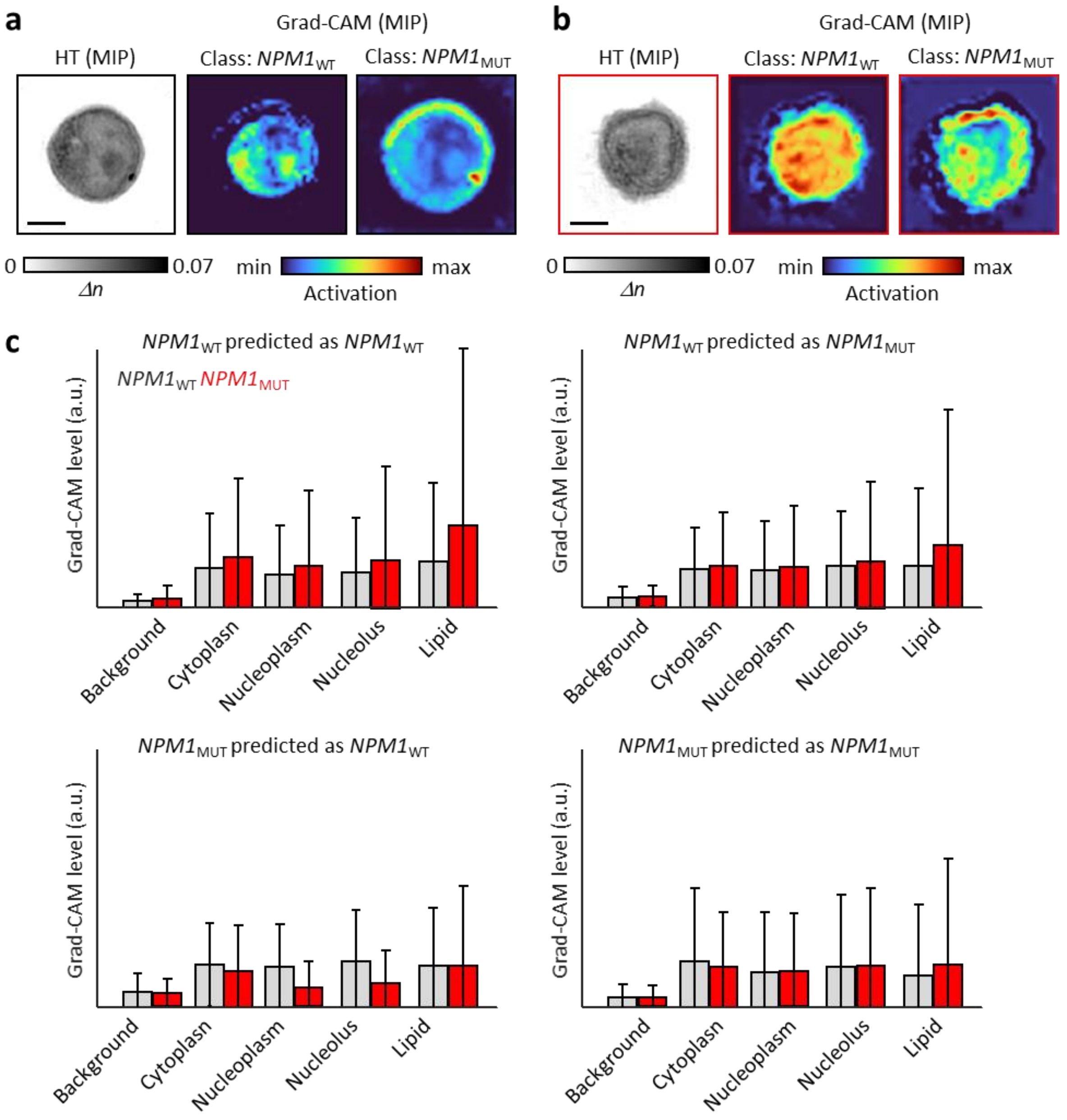

Supplementary Figure S2. Comparative study of classifier network size. The classification accuracy decreases with the reduction of network parameters.

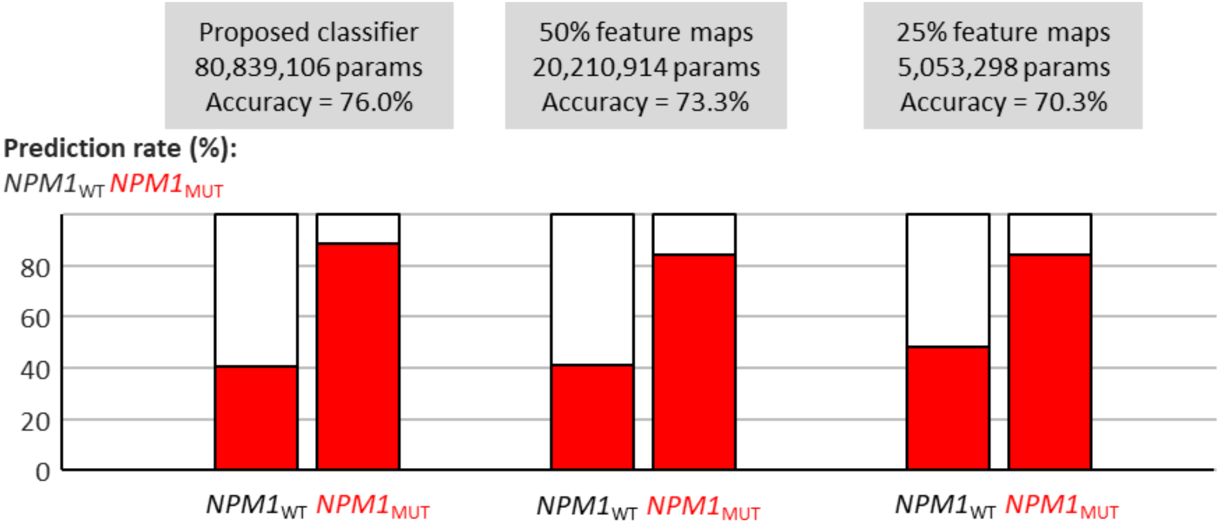

Supplementary Figure S3. Ablation study regarding image resolution. Numerically reducing the resolution decreases the classification accuracy.

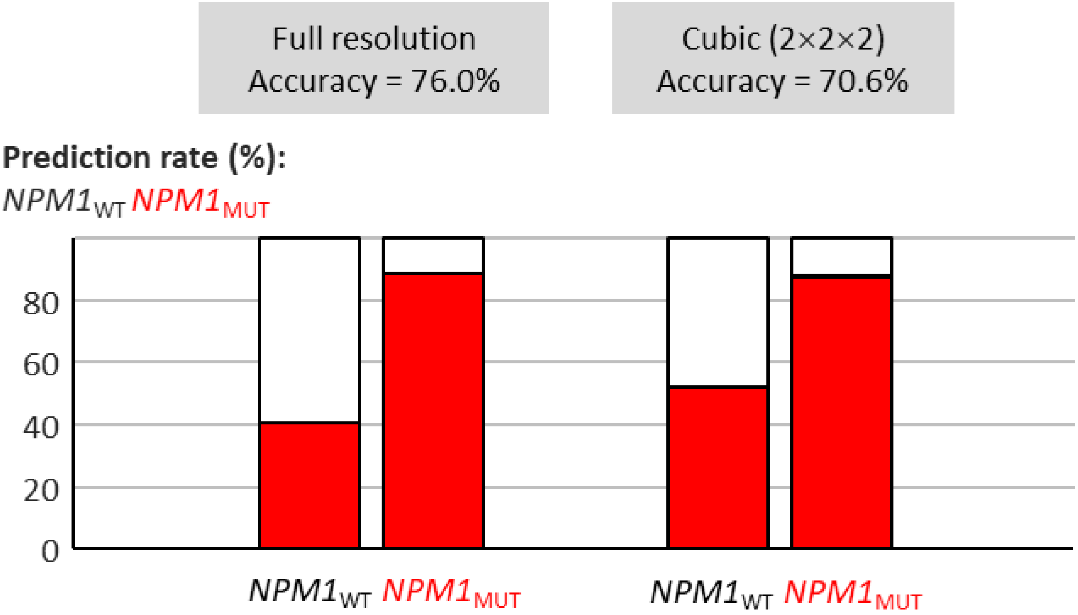

Supplementary Figure S4. Ablation study regarding the number of data in training. (a) Graphical description of the data options used in the ablation study. The number of data directly used in the optimization are altered. (b) The comparison of the accuracy indicates the significance of data scale in the classifier performance.

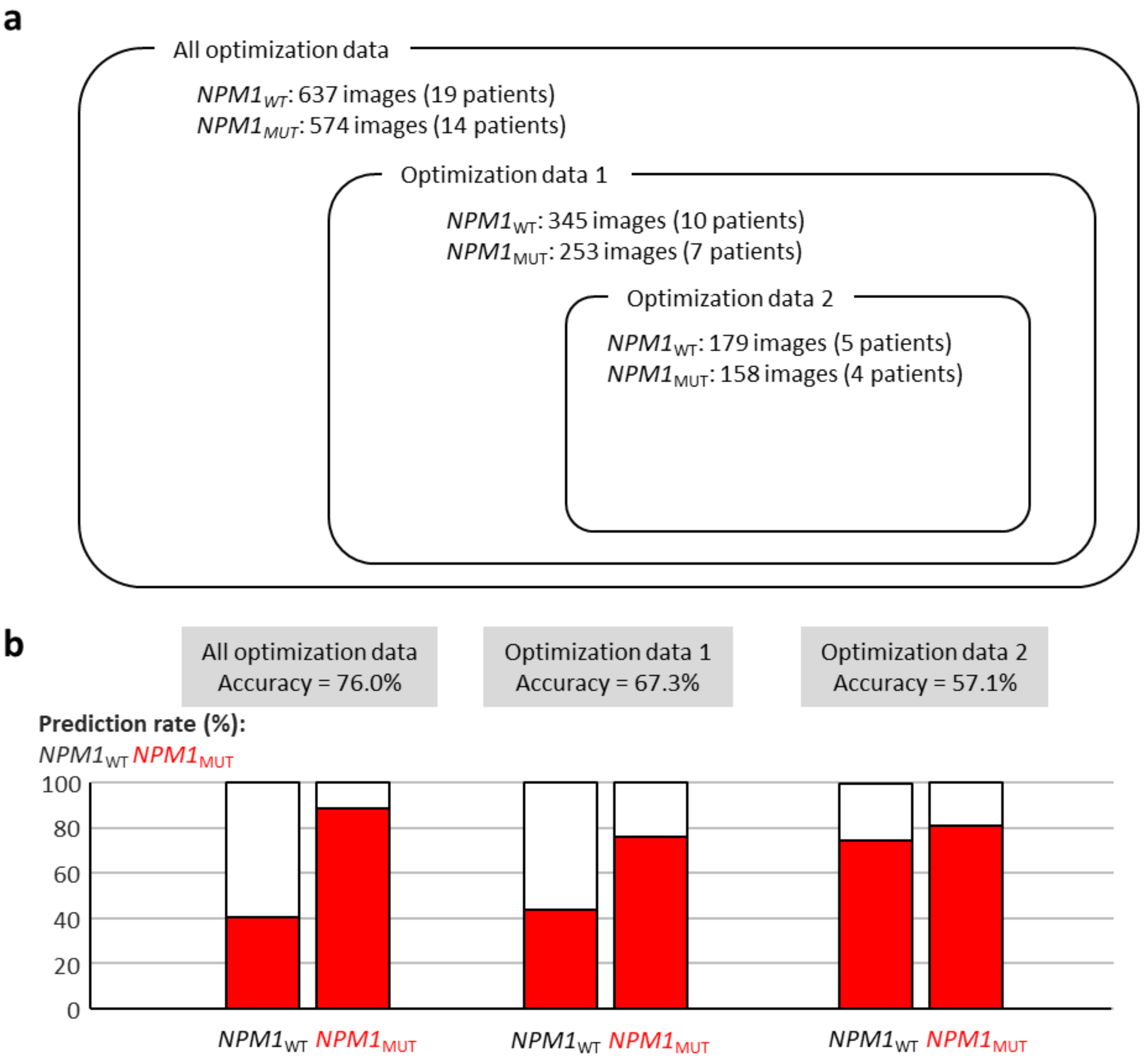

Supplementary Figure S5. Pictorial description of the network structure. (a) The overall structure of our FishNet-inspired 3D image classifier. The multipliers indicate the number of residual blocks included in each unit. The stem unit consists of three convolutions, the first of which with a stride of 2, and a max pooling operation 2×2×2. Every convolution in the network is followed by an instance normalization and a leaky rectified linear unit. (b) The composition of a down-refinement unit. (c) The composition of an up-refinement unit. (d) The structure of a residual block, which is repeatedly used in the implementation.

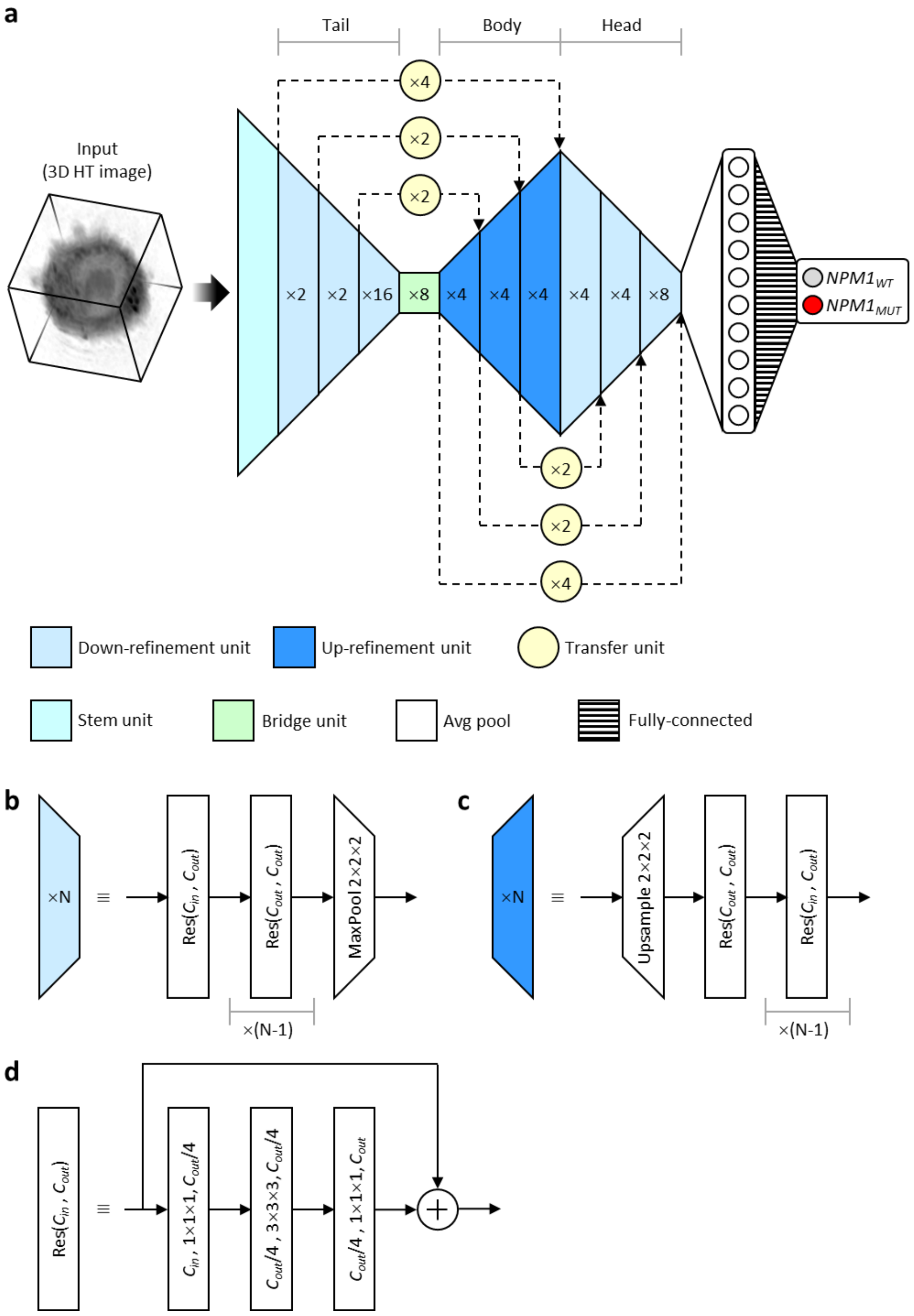

Supplement: Supplementary file 1 — Supplementary Information. [file 41598_2024_75168_MOESM1_ESM.pdf]
